# Breakthrough in Luminescence Thermometry—Supersensitive Emission Line Shift of Whispering Gallery Modes in Rhodamine B‑Doped Cellulose Fiber Microresonators

**DOI:** 10.1021/acsami.5c12765

**Published:** 2025-10-01

**Authors:** Przemysław Woźny, Kevin Soler-Carracedo, Małgorzata Skwierczyńska, Inocencio R. Martin, Piotr Kulpiński, Marcin Runowski

**Affiliations:** † Faculty of Chemistry, Adam Mickiewicz University, Uniwersytetu Poznańskiego 8, Poznań 61-614, Poland; ‡ Departamento de Física, Instituto de Materiales y Nanotecnología (IMN), Universidad de La Laguna, San Cristóbal de La Laguna E-38200, Santa Cruz de Tenerife, Spain; § Department of Mechanical Engineering, Informatics and Chemistry of Polymer Materials, Lodz University of Technology, Żeromskiego 116, Łódź 90-924, Poland

**Keywords:** optical microresonator, temperature sensing, luminescent thermometer, cellulose microfibers, organic dyes

## Abstract

The development of optically active materials enabling rapid, precise, and accurate remote detection of physical parameters is crucial for advancing science and modern technology. In this work, we investigate resonant effects and light propagation in cellulose fibers doped with Rhodamine B for optical thermometry applications. These fibers were successfully produced by using the spinning method with *N*-methylmorpholine N-oxide. Their optical properties were investigated through absorption and emission spectroscopy, confirming the integration of Rhodamine B into the cellulose matrix. Notably, the cylindrical shape of the modified fibers significantly affects the emission spectra when excited at the fiber edge, revealing sharp and superimposed whispering gallery modes (WGMs). A confocal system with a 532 nm laser was used to analyze for the first time the WGM emission from the optically active cellulose microfibers. The WGMs displayed high susceptibility to the negative thermo-optical coefficient of the resonating cavity, leading to a giant spectral shift. This unprecedented temperature-induced blue shift of the WGMs provides the highest reported sensitivity-27 times higher than other microresonators—demonstrating a spectral shift of ∼0.47 nm K^–1^. With excellent temperature resolution (≈0.17 K), our findings highlight the great potential of this method and material as a supersensitive optical thermometer.

## Graphical abstract

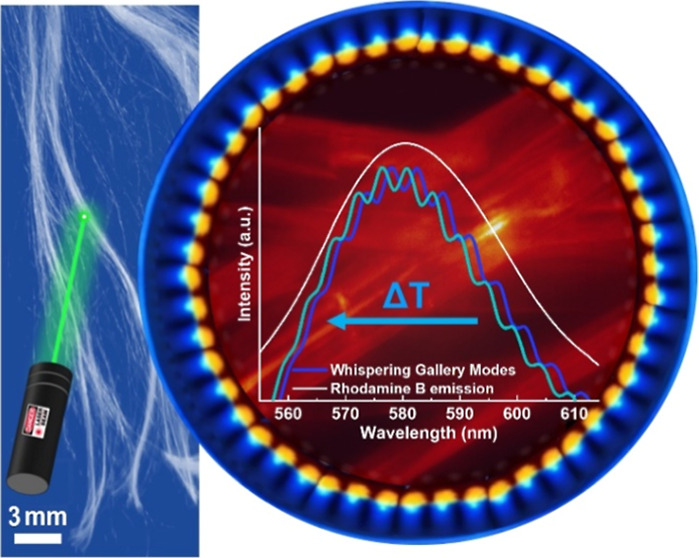

## 1 Introduction

The research on new optical materials is required for the development of precise, highly accurate optical temperature sensors, which are essential for various applications, e.g., for industrial temperature monitoring, medical diagnostics, or space technology.^1–4^ Different approaches for contactless temperature measurements, mainly based on luminescence intensity ratio (LIR) and emission lifetimes of inorganic materials doped with lanthanide or d-block metal ions, along with multiparameter sensing, are essential for technological advancement.^5–9^ By enhancing the sensitivity and stability of these sensors, one can achieve more accurate and reliable temperature readouts. As a result, the ongoing research focuses mainly on inorganic optical materials, which suffer from low sensitivity of their emission line shift to temperature variations, which is the simplest and most convenient approach in optical detection.

On the other hand, cellulose fibers are polysaccharide materials combined with the β-1,4-glucose units in a linear and long chain. Cellulose fibers offer a promising platform for a wide range of applications due to their flexibility and abundant availability. The beneficial factor of cellulose fibers is their ecological and biodegradable character.^10,11^ Moreover, they can be easily obtained via various approaches, e.g., the viscose method, Lyocell method, acetate method, and dry-wet and dry spinning processes.^12–17^ In recent years, appropriately functionalized, optically active fibers have gained significant interest in the fields of photonics and sensing technologies. Doping cellulose fibers with additives, such as organic dyes, quantum dots, or magnetic or plasmonic nanoparticles, allows one to achieve unique optical, magnetic, or plasmonic properties without drastic changes in their mechanical properties.^18–22^ Among various functionalizations explored, doping cellulose fibers with fluorescent dyes has emerged as a compelling strategy to enhance their optical properties.^23–27^

Rhodamine B is a well-known organic dye with excellent luminescence properties, i.e., bright orange-red emission, high quantum yield, and good stability.^28–30^ Doping cellulose fibers with Rhodamine B benefits specific photoluminescence properties upon excitation with an external source of radiation. Development of materials doped with Rhodamine B, in contrast to semiconducting quantum dots and dielectric inorganic nanoparticles (based on lanthanides or d-block metal ions), is cheaper, consumes less energy, is bidegradable, and helps to avoid the introduction of heavy metal ions into the environment. Moreover, the organic structure of Rhodamine B is resistant to temperatures below ≈500 K, allowing the use of this compound as a modifier of cellulose fibers in cryogenic, biological, and high-temperature ranges, which is beneficial for temperature sensors working in diverse applications.^31,32^

Microresonators are micron-sized optical structures that can trap the radiation within their periphery through continuous total internal reflection around the material when the radiation waves propagate along the inner boundary without escaping.^33^ That effect is possible in the materials with spherical, disk-shaped, cylindrical, or toroidal morphology, e.g., microspheres, microfibers, etc.^34^ Microresonators are well-known due to their ability to exhibit the whispering gallery modes (WGMs) superimposed on their emission spectra, i.e., the regularly shifted, intense narrow peaks as a result of resonance after internal reflection of the radiation by the material–air interface due to differences in the refractive indices. The potential applications of microresonators are widely investigated as optical sensors of temperature, pressure, and chemical species due to the high sensitivity of WGM shift to environmental changes.^35–39^

Remote, luminescent thermometers are extensively studied materials because they can be used in systems and media that are unavailable for classical thermometers. Optical temperature sensors have many advantages over traditional thermometers, such as electrical passiveness, lack of electromagnetic interference, rapid readout, better sensitivity, greater dynamic range, etc.^5^ Most of the commonly used materials in optical thermometry are inorganic materials doped with lanthanide ions (i.e., Pr^3+^, Er^3+^, Tm^3+^, Nd^3+^), due to their narrow emission bands and ease in the determination of the luminescence intensity ratio (LIR) between bands.^40–42^ On the other hand, using microresonators for optical temperature sensing can also be done remotely, and it was previously investigated in some materials (mainly inorganic microspheres) doped with Ho^3+^, Er^3+^, or Nd^3+^.^39,43,44^ Rhodamine B has a short luminescence decay time (τ ∼ 3 ns) in comparison to Ln^3+^ ions (range of μs to ms) and is well-known for its high quantum yield (Φ ∼ 0.59). Such high luminescence efficiency and the short lifetime allow for rapid readouts with a low noise-to-signal ratio.^45^ The spectral shift of the luminescence resonator emission peaks—WGMs—is primarily caused by two factors, i.e., thermal expansion of the material and changes in optical properties, specifically the refractive index (*n*) and, therefore, the thermo-optic coefficients (d*n*/d*T*) of the material.^34,44,46,47^

Here, cellulose fibers modified with Rhodamine B (cellulose fiber@Rhodamine B) were fabricated using the Lyocell method with *N*-methylmorpholine N-oxide (NMMO) as a solvent. The fibers were obtained by a dry-wet spinning technology. Using a modified confocal setup (Figure 1), we examined for the first time the great potential of the luminescent cellulose fibers doped with fluorescent dye Rhodamine B as optical temperature sensors based on the WGM shift. It turned out that the developed cellulose fiber-based WGM temperature sensors exhibit unprecedentedly high thermal sensitivity (0.47 nm K^–1^), which is over 27 times better compared with any luminescent microresonators operating in a band-shift mode.

**Figure 1:**
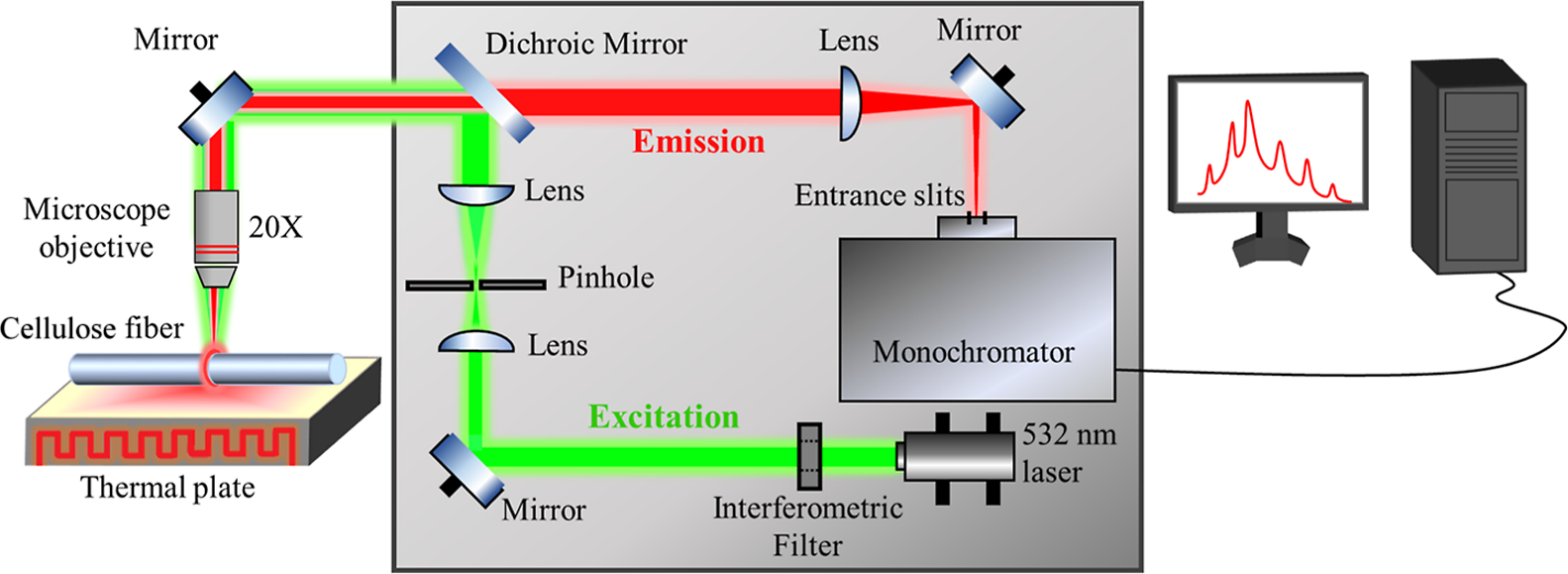
Scheme of the experimental, confocal setup used to measure the temperature-dependent emission spectra with WGMs, originating from the cellulose fibers functionalized with the Rhodamine B.

## 2 Experimental Section

### 2.1 Synthesis and Fabrication

The cellulose solution was prepared using a pulp from Rayonier (polymerization degree (DP) 1250, 96.8 wt % % of α cellulose) and 50% aqueous solution of NMMO (from Huntsman Holland BV, The Netherlands). To stabilize the molecular weight, the propyl ester of gallic acid Tenox PG (purchased from Aldrich, Gillingham, Dorset, UK) was applied as an antioxidant.

In order to obtain the spinning dope, an appropriate amount of cellulose pulp with a 50% aqueous solution of NMMO and Tenox PG (1 wt % calculated for a-cellulose content) was placed in the IKAVISC kneader. Afterward, cellulose was vigorously stirred under low pressure (≈20 hPa) and heated to 388 K. The process was carried out for 1.5 h until the water content in the system did not exceed 14 wt % and a homogeneous solution of cellulose was obtained. The resulting spinning dope was placed into a preheated cylinder (temperature not exceeding 338 K). Subsequently, the solution was pressed by a piston through the nozzle holes and solidified in a coagulation bath containing cold water. The fibers were spun at a take-up speed of 50 m/min, washed, and dried at room temperature (RT). The cellulose fibers were immersed in 1 mL of 10^–4^ M ethanol solution of Rhodamine B, and then the cellulose fibers were air-dried.

### 2.2 Characterization

Absorption spectra were recorded with a JASCO V-770 spectrophotometer equipped with a spherical integrator ILN-925 with detectors: a photomultiplier in the UV–vis range (λ = 200–850 nm) and a PbS detector in the NIR range (λ = 850–1200 nm), and two radiation sources: the deuterium (λ = 190–340 nm) and the halogen lamps (λ = 340–1200 nm). X-ray diffraction (XRD) patterns were recorded using a Bruker AXS D8 Advance diffractometer in Bragg–Brentano geometry with Cu Kα1 radiation (λ = 1.5406 Å) and a 0.05° step. The reference pattern data were taken from ICSD (Inorganic Crystal Structure Database). The SEM images and elemental mapping were done with a scanning electron microscope, FEI Quanta 250 FEG, equipped with an EDAX detector. Photos of the cellulose fibers were collected with a Nikon D3000. The excitation spectrum was investigated using a Hitachi F7000 spectrofluorometer equipped with a 150 W xenon lamp. All emission spectra were recorded using an Andor SR-500i-B2 spectrometer coupled with a CCD camera Newton 970EMCCD. Modified temperature conditions were obtained with the heating–cooling stage Linkam THMS600 with a temperature stability of 1 K. Emission spectra with WGMs were recorded in the confocal setup described using the Renishaw InVia confocal micro-Raman system with a silicone-based CCD camera as a detector. Emission spectra were obtained by exciting the material with a continuous-wave 532 nm diode-pumped 100 mW solid-state laser with a focal point of ∼20 μm^2^. For the excitation and detection, a confocal microscope setup with an Olympus ×20 SLMPlan N long working distance objective was used (schematically shown in Figure 1). All spectra were corrected for the spectral response of the equipment. To calibrate the sensor with the temperature, the fibers were located inside a chamber of a heating–cooling stage. The integration time used was 0.5 s.

To obtain the spectral resolution of the setup, the width of a single-mode laser line at 532 nm was measured at RT. The resulting spectral resolution was approximately 140 pm, being in good agreement with the manufacturer’s specifications. For the resolution limit of the system, 100 measurements of a laser profile under the same conditions were recorded. The spectra were fitted to a Gaussian curve, and the uncertainty was calculated as the standard deviation of the laser peak position distribution. A standard deviation of 3 pm was obtained. A similar procedure was carried out to obtain the uncertainty of the sensor, analyzing the spectral position of a single resonant mode at RT for 100 measurements. The double standard deviation obtained from the statistical analysis was used as the error for the spectral position of the WGM, whereas the typical error of a type K thermocouple was used for the error in the temperature axis.

## 3 Results and Discussion

Figure 2a shows chemical structures of the xanthene-based organic dye Rhodamine B and cellulose, i.e., organic components used to fabricate the fluorescent microresonators based on organic dye-modified cellulose fibers. The crystal structure of the cellulose fibers doped with Rhodamine B (cellulose fiber@Rhodamine B) was investigated with the powder XRD method and compared with plain cellulose fibers. The recorded XRD pattern of the cellulose fibers shows a small peak at 2θ values of ∼12.3° and one intense peak at around 21.1°, as a result of overlapped reflexes at ∼19.3° and 22.0°, caused by the low crystallinity and partially amorphous structure of the fabricated cellulose in the form of fibers (Figure 2b). Both reflexes are assigned to the (110) and (020) *hkl* planes of type II cellulose.^48^ The distorted intensity ratio (compared to the ref pattern) between reflexes at ∼20° and 41° is a result of the fiber-shaped growth of cellulose, resulting in changes in the plane ratios.^49^ There are no additional reflexes from Rhodamine B due to its small content and negligible influence of the organic Rhodamine B molecules on the internal structure of the examined material.

**Figure 2:**
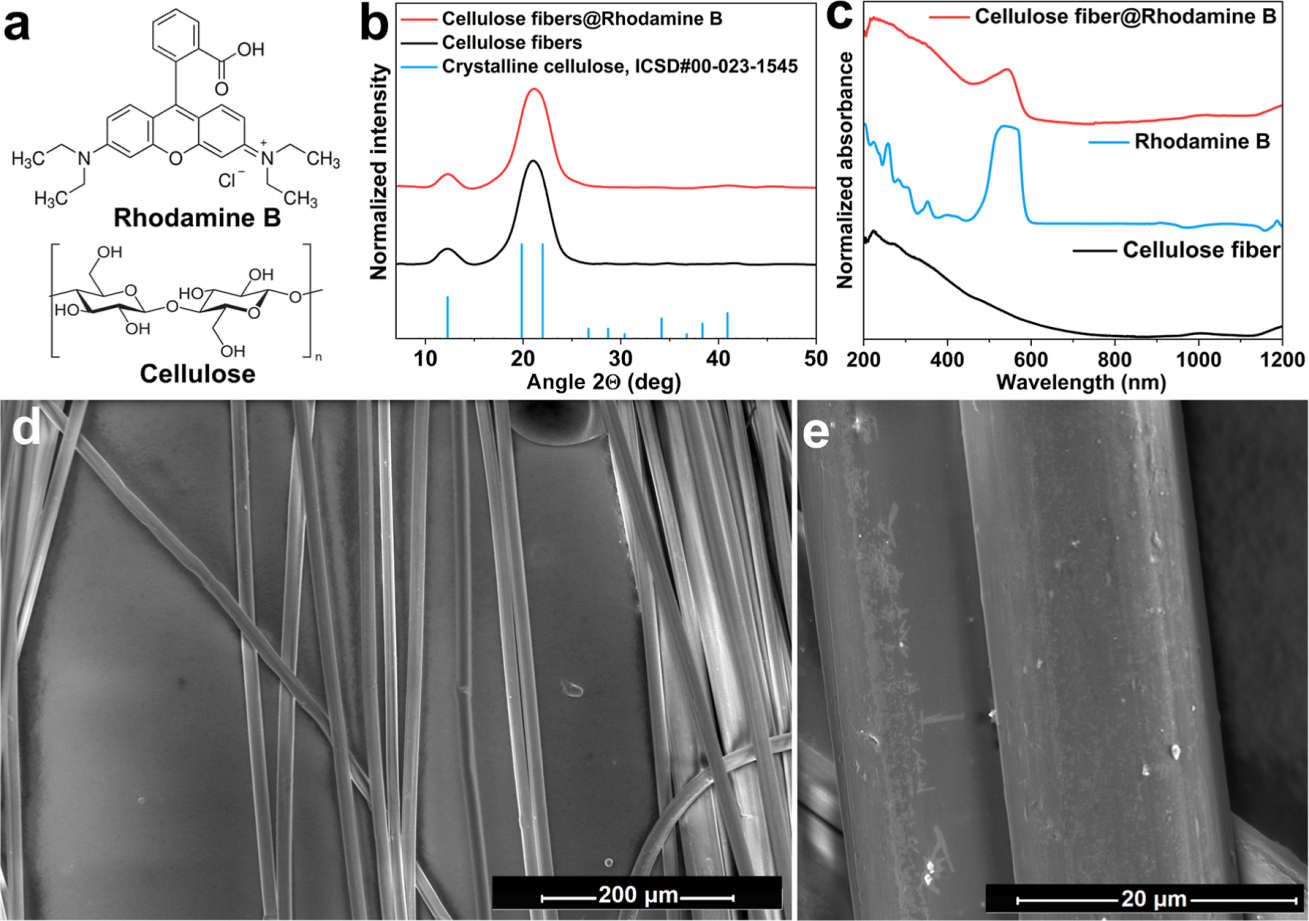
Chemical structure of Rhodamine B and cellulose (a); XRD patterns of the bare and Rhodamine B-modified cellulose fibers (b); absorption spectra of the plain cellulose fibers, Rhodamine B, and Rhodamine B-doped cellulose fibers (c); SEM images of the cellulose fiber@Rhodamine B (d,e).

Absorption spectra for the unmodified cellulose fibers, the organic dye Rhodamine B, and the cellulose fibers doped with Rhodamine B were recorded and are compared in Figure 2c. The spectrum of Rhodamine B shows high absorbance in the range of 200–300 nm and an intense absorption band at around λ ≈ 480–580 nm, allowing effective absorption of the green laser radiation at λ = 532 nm. The plain cellulose fibers obtained with the Lyocell method show a broad absorption band, ranging from UV (λ ≈ 200 nm) with a gradual decrease of absorbance up to 700 nm, as well as two small bands in the NIR range at λ ≈ 900–1300 nm.^50^ Rhodamine B-doped cellulose fibers present an absorption spectrum similar to that of unmodified cellulose fibers with an additional, strong absorption band with a maximum at λ = 542 nm, characteristic of the intense absorption band of Rhodamine B. SEM images of the cellulose fiber@Rhodamine B (Figure 2d,e) confirm their homogeneity and uniformity, with a diameter of ∼16 μm (±1.7 μm).

Figure 3a–c presents photographs of the synthesized cellulose fibers doped with Rhodamine B in daylight (a, b) and their bright red photoluminescence under UV light radiation (c). This is because Rhodamine B, as a fluorescent dye, upon UV irradiation undergoes a π → π* electronic transition, being excited from the highest occupied molecular orbital (HOMO) in its ground state (S_0_) to the lowest unoccupied molecular orbital (LUMO) in the first excited singlet state (S_1_).^51^ Subsequently, the fluorescence process occurs as the molecule relaxes from the S_1_ to the S_0_ state, emitting visible photons. The detailed mechanism of the Rhodamine B emission is presented in the Supporting Information (Figure S1a). In order to investigate the optical properties of the functionalized materials in detail, the emission and excitation spectra of the Rhodamine B crystals, Rhodamine B solution (in ethanol), and cellulose fibers@Rhodamine B were initially recorded (Figure 3d). The obtained excitation spectra (black curves) show well-structured, intense, and broad bands of Rhodamine B, whose altered shape reveals different natures of chemical interactions between the organic molecules in solid and liquid forms, such as electrostatic attraction/repulsion, dimerization, π–π stacking, etc.^30,52–54^ However, the emission spectra (green, blue, and red, respectively) exhibit two intense, broad, and overlapping bands, with maxima at around 700 and 720 nm for the stacked dimer structure in solid crystals, 600 and 620 nm for the monomer–dimer structure in solution, and 590 and 620 nm for the monomer structures conjugated to the cellulose polymer, which are related to the visible orange-red fluorescence of Rhodamine B and agree well with the literature data.^30,52–54^

**Figure 3:**
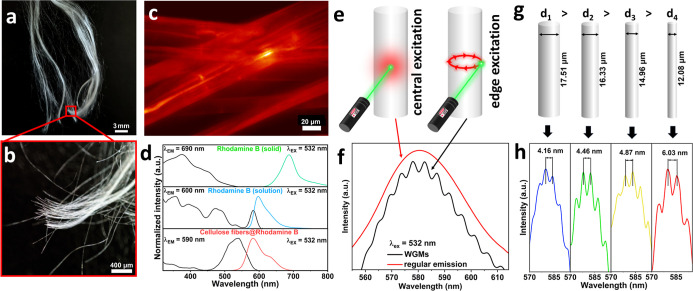
Photographs of the Rhodamine B-modified cellulose fibers in daylight (a,b) and under UV excitation (c); normalized excitation and emission spectra of the solid Rhodamine B, its solution in ethanol, and cellulose fibers modified with Rhodamine B (d); a schematic representation of the excitation geometry for regular (left; central excitation and detection) and WGMs (right; edge excitation and detection) emissions (e); regular luminescence (red curve) and WGM (black curve) emission spectra from the cellulose fiber doped with Rhodamine B upon 532 nm laser excitation (f); schematic representation of fibers with different diameters (g) and measured WGM emission spectra with experimentally determined FSR values (h).

In order to investigate the optical activity of the cellulose fibers modified with Rhodamine B, we used a modified confocal microscopy setup (see Figure 1), allowing geometry adjustments of the optical path, excitation beam, and detection line. Figure 3e depicts a visualization of the conventional luminescence vs the WGM emission phenomenon, depending on the excitation and detection geometry, i.e., central excitation–detection for conventional emission vs edge excitation–detection for WGM emissions. Excitation of the dye-doped fibers in the center of the fiber at an angle of 90° (Figure 3e; left) with a 532 nm laser results in the regular emission spectrum of Rhodamine B (Figure 3f; red curve). As was already mentioned, when measuring the emission spectra for the cellulose fiber@Rhodamine B using a confocal microscope setup, it is possible to modify the position of the excitation beam and detection geometry, setting them to an edge of the cellulose fiber when the incident angle of the laser beam decreases (Figure 3e; right), and observe the effect of WGMs (Figure 3f; black curve). In such a case, the initially broad emission band related to Rhodamine B fluorescence drastically changes, showing a series of superimposed narrow, sharp peaks, i.e., WGMs, as clearly seen in Figure 3f. The spectral position of these resonant modes can be described by using the optical-ray approximation with eq 1:^35^

1 $$\lambda_{m}=\frac{2\pi }{m}\cdot n_{eff}R$$

where *m* is the mode number, λ_*m*_ is the resonant wavelength, *n*_eff_ represents the effective refractive index, and *R* is the radius of the microresonator.^55,56^ The free spectral range (FSR) of the resonant modes depicted as Δν, i.e., the distance between the individual WGM peaks in the spectrum, is usually described by eq 2:^57^

2 $$\Delta \nu =\frac{c}{2\pi n_{eff}R}$$

where *c* is the speed of light in vacuum, *n*_eff_ is the effective refractive index, and *R* is the radius of the circular light path in a microresonator. As shown in eq 2, the radius of the fiber influences the FSR in the emission spectrum with whispering gallery modes (Figure 3g,h). We investigated four individual cellulose fibers with varying diameters (17.51, 16.33, 14.96, and 12.08 μm) to compare the WGMs and FSR. As can be seen in Figure 3h, the decrease in the microresonator radius leads to a greater separation of the individual modes observed in the WGM spectrum, i.e., 4.16, 4.46, 4.87, and 6.03 nm, respectively. Therefore, for precise detection of WGM positions with high accuracy, it is advantageous to use fibers with smaller diameters, as they exhibit a higher FSR factor.^47^ However, there is a drawback that should be considered here. The WGM spectral position can be affected by environmental changes in the refractive index, and while this effect may be neglected in most cases, it becomes more relevant with decreasing resonator diameter.^58,59^ In the cases of real applications where the microresonator will be located in a medium susceptible to changes in its refractive index, an agreement in the size of the fiber should be reached in order to optimize the FSR factor and minimize the errors due to environmental refractive index changes.

Additionally, we estimated the approximated effective refractive index value, based on eq 2 and the experimentally obtained values of the radius of the fibers and corresponding FSR, resulting in *n*_eff_ = 1.444 ± 0.003. Using this value, we simulated the evolution of FSR values as a function of the fiber diameter and overlaid the obtained curve with the experimental data points (Figure S1b). We consider that, particularly from the application point of view, predicting the distance between the WGMs based on the fiber diameter could be useful for optimization and adjustment of the temperature-sensing performance.

Afterward, the optical properties of the Rhodamine B-doped cellulose fibers, i.e., their emission spectra superimposed with WGMs, were investigated at variable temperature conditions (Figure 4a). Under elevated temperature conditions, all WGMs show a blue shift (hypsochromic) (Figure 4b). The determined spectral positions (peak centroids) for selected WGMs as a function of the temperature are presented in Figure 4c. The WGM spectral position shifts can be described using the following equation:^55^

3 $$\frac{d\lambda_{m}}{\lambda_{m}}=\frac{dn_{eff}}{n_{eff}}+\frac{dR}{R}$$

indicating that the variation in the WGM spectral position is related to the changes in the effective refractive index and the radius of the optical resonator. When these variations are induced by temperature changes of the system, the observed effects (WGM positions) can be described by the modified formula (eq 3):^55^

4 $$\frac{d\lambda_{m}}{\lambda_{m}}=dT\left(\frac{1}{R}\frac{dR}{dT}+\frac{1}{n_{eff}}\frac{dn_{eff}}{dT}\right)=dT\left(\alpha +\beta \right)$$

where α and β are the thermal expansion and thermo-optic coefficients, respectively. Photophysical changes taking place in the fibers under elevated temperature conditions, i.e., change in the refractive index, increase of their diameter, and WGM shift, are schematically presented in Figure 4b.

**Figure 4:**
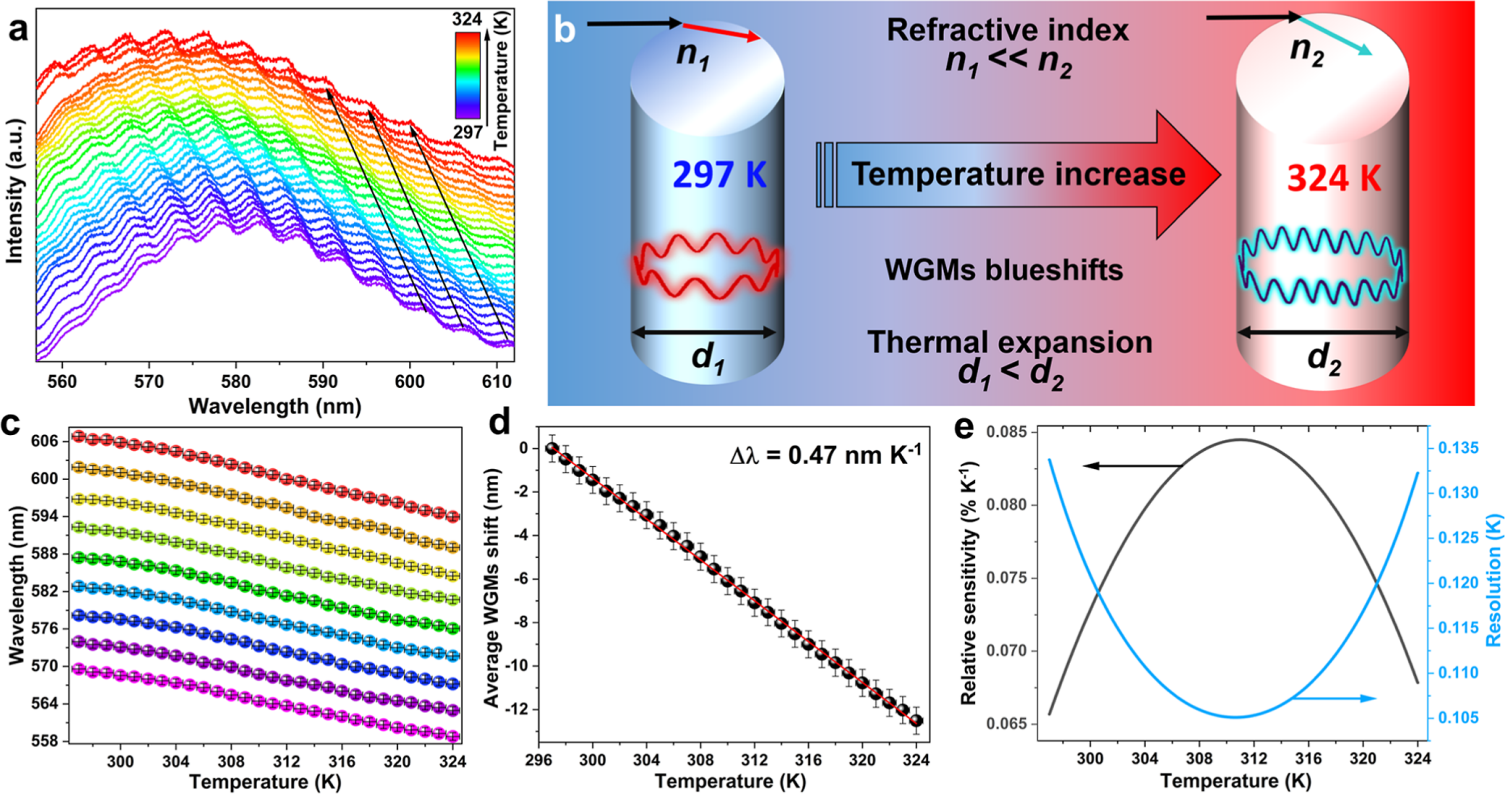
Emission spectra of Rhodamine B-doped cellulose fibers measured at 532 nm laser excitation at different temperature values, showing the appearance of narrow WGMs and their thermal shift (a); scheme of the thermal expansion of the cellulose fibers (b); WGM spectral positions as a function of temperature (c); determined average displacement of the WGM as a function of temperature fitted to a linear function, indicating their spectral blue shift (d); relative sensitivity (black curve; left axis) and determined temperature uncertainty—thermal resolution (blue curve; right axis) of the temperature sensor based on the WGM spectral shift of cellulose fiber@Rhodamine B (e).

In the obtained modified fibers, the resulting blue shift is due to the great influence of the negative value of the thermo-optic coefficient and the marginal effect of the thermally induced expansion of the material. It is worth noting that in the systems exhibiting spectral red shift of the WGMs, the thermal expansion effect plays a dominant role.^60^ However, in our case, the thermal expansion coefficient for the cellulose fibers is low, and it has a value of around ∼5 × 10^–6^ K^–1^, being 2 orders of magnitude smaller than their thermo-optic coefficient (around −1 × 10^–4^ K^–1^), similar to other polymer materials.^46,61–64^ Therefore, thermal expansion has minor importance, compared to the thermo-optic coefficient, when deciding on the choice of cellulose fibers as the host material for novel optical sensors based on the WGM phenomenon. Hence, given the negative value of the thermo-optic coefficient of cellulose, a spectral blue shift of the WGM is expected when the fiber is heated, as experimentally confirmed in Figure 4a,c.^61–63^ It is worth noting that a temperature change does not significantly affect the optical properties of air; the temperature-induced change of its refractive index is negligible. This is because the thermo-optic coefficient of air (d*n*/d*T* ≈ −0.55 × 10^–6^ K^–1^) is 2 orders of magnitude smaller than that of cellulose fibers.^65,66^

Figure 4d presents the average temperature-induced shift of WGMs for the 580 nm band, from 297 K (RT) up to 324 K. For a temperature increment of 27 K, the WGM peak shifts by approximately 12.5 nm. The obtained data points of the WGM spectral position as a function of temperature were fitted to a linear function (empirical model) using eq 5:

5 Δλ=139.5536−0.4697×T

where Δλ is an average shift and *T* is the temperature of the system. Taking into account that the spectral shift of the WGM with temperature is associated both with the thermal expansion and thermo-optic coefficients of the resonator,^67^ the linear dependence observed in Figure 4d indicates a linear change of the thermo-optic coefficient in the specified temperature range, assuming that the thermal expansion of the material is linearly dependent on temperature (which is valid in the case of the absence of phase transitions). As the spectral shift of average WGM shift is represented by the linear function, the slope value of the curve represents the absolute sensitivity (*S*_a_) of the cellulose fibers@Rhodamine B microresonator, i.e., Δλ ≈ 0.47 nm K^–1^.

To characterize the temperature-sensing properties of the material, the relative sensitivity (*S*_r_) was calculated according to eq 6:^40^

6 $$S_{r}=100\%\frac{1}{M_{p}}\left|\frac{dM_{p}}{dT}\right|$$

where *M*_p_ is the measured parameter, i.e., the WGM spectral position. The temperature dependency of the *S*_r_ value was determined based on the fitted curve of an average shift of WGMs from Figure 4d (i.e., calculated WGM positions as a function of temperature) and is presented in Figure 4e (left axis). The *S*_r_ parameters significantly increase with the temperature of the system up to 312 K, where a plateau is reached (*S*_r_ = 0.084% K^–1^) and a reversed tendency is observed, i.e., a slight and gradual decrease with further temperature elevation. The photostability of the obtained material was investigated by recording 20 emission spectra with 1 s intervals under continuous excitation by a 532 nm laser, resulting in the luminescence intensity decreasing only by ∼3% (Figure S2a). Additionally, the performed studies demonstrate that prolonged exposure to the excitation laser beam does not cause any detectable shift of the superimposed WGMs, confirming the absence (or negligible) of laser-induced optical heating. To prove the accuracy, repeatability, and reliability of the obtained results and developed sensor, we have measured the average displacement of the selected WGMs in 5 heating–cooling cycles (see Figure S2b). The presented results clearly indicate the repeatability of temperature-sensing measurements with cellulose fibers modified with Rhodamine B. Furthermore, we investigated the temperature dependence of the WGMs for the second fiber to confirm the obtained results. The results of the second run of measurement, i.e., the emission spectra with visible WGMs, the calculated mode positions, and the average WGM shift, are shown in Figure S3a–c, respectively. Another important parameter used to characterize optical sensors is the limit of detection (LOD), also known as sensing resolution—δ*T* (uncertainty).^40,68^ The value of sensing resolution is related to the minimum change of the parameter that the sensor is capable of detecting. It can be theoretically calculated using the formula given below (eq 7):^40^

7 $$\delta T=\frac{1}{S_{r}}\times \frac{\delta M_{p}}{M_{p}}$$

where δ*M*_p_/*M*_p_ is the relative uncertainty of determination of the measured parameter (in this case, the WGM spectral position error). The δ*M*_p_ depends mostly on the experimental setup, spectral resolution, acquisition time of the signal, and temperature-dependent shift. Here, we have calculated the theoretical evolution of the δ*T* as a function of temperature (Figure 4e; right axis), based on the determined *S*_r_ parameter. The δ*T* is ∼0.133 at 297 K, and it decreases to around 0.105 at 310 K and starts to increase further with temperature elevation up to 0.132 at 324 K. The second approach is based on the determination of the experimental temperature uncertainty by collecting a series of 100 spectra, subsequent measurements of WGM position for each case, and the calculation of the statistical distribution of those values. Here, we determined at RT and presented the LOD for two parameters: WGM spectral position and temperature in Figure S4a,b, respectively. The lowest possible change of the spectral position of WGMs was calculated as 0.057 nm, resulting in an LOD of 0.17 K, which is quite similar to the calculated theoretical value, indicating very good temperature-sensing resolution for the developed optical thermometer. For real-time temperature monitoring of biological processes, the target of LOD as 0.1 K at 0.1 s readout (0.1 s × 0.1 K) is desired.^69^ Here, we have estimated that under the applied measuring conditions, our material exhibits thermal and temporal resolution of 0.085 K s, which implies that the developed method of optical temperature sensing with the band shift of the superimposed WGMs needs further investigation for real-time temperature monitoring in biological systems.

The constant development of technology requires the use of advanced research equipment that enables precise material analysis for cutting-edge technologies. The presented research indicates the need for a refined method employing sophisticated confocal equipment, which allows material excitation and the recording of emission spectra from superimposed WGMs at a precise point of the material, the edge of a micrometric cellulose fiber. However, despite this barrier, this approach enables highly sensitive temperature detection. The determined *S*_a_ and *S*_r_ values allowed us to compare the developed optical sensor with other materials exhibiting WGM effects (see Table S1). The obtained results show that our material truly exhibits the best performance, i.e., the highest absolute (27 times better) and relative sensitivities (42 times better) among all reported luminescent thermometers based on the microresonators with WGMs studied so far. The unprecedentedly high value obtained for the absolute and relative sensitivities in the physiological temperature range suggests the potential use of this material as a supersensitive optical thermometer for advanced biomedical applications requiring very high accuracy of temperature monitoring. Furthermore, besides the materials exhibiting WGMs, the calculated absolute sensitivity was compared with other luminescent materials, i.e., the most sensitive (band shift-based) optical thermometers based on organic dyes or inorganic phosphors reported to date (Figure 5). This comparison demonstrates an exceptional sensitivity (twice better than the most sensitive optical thermometer reported) and great application potential of the temperature sensor we developed.

**Figure 5:**
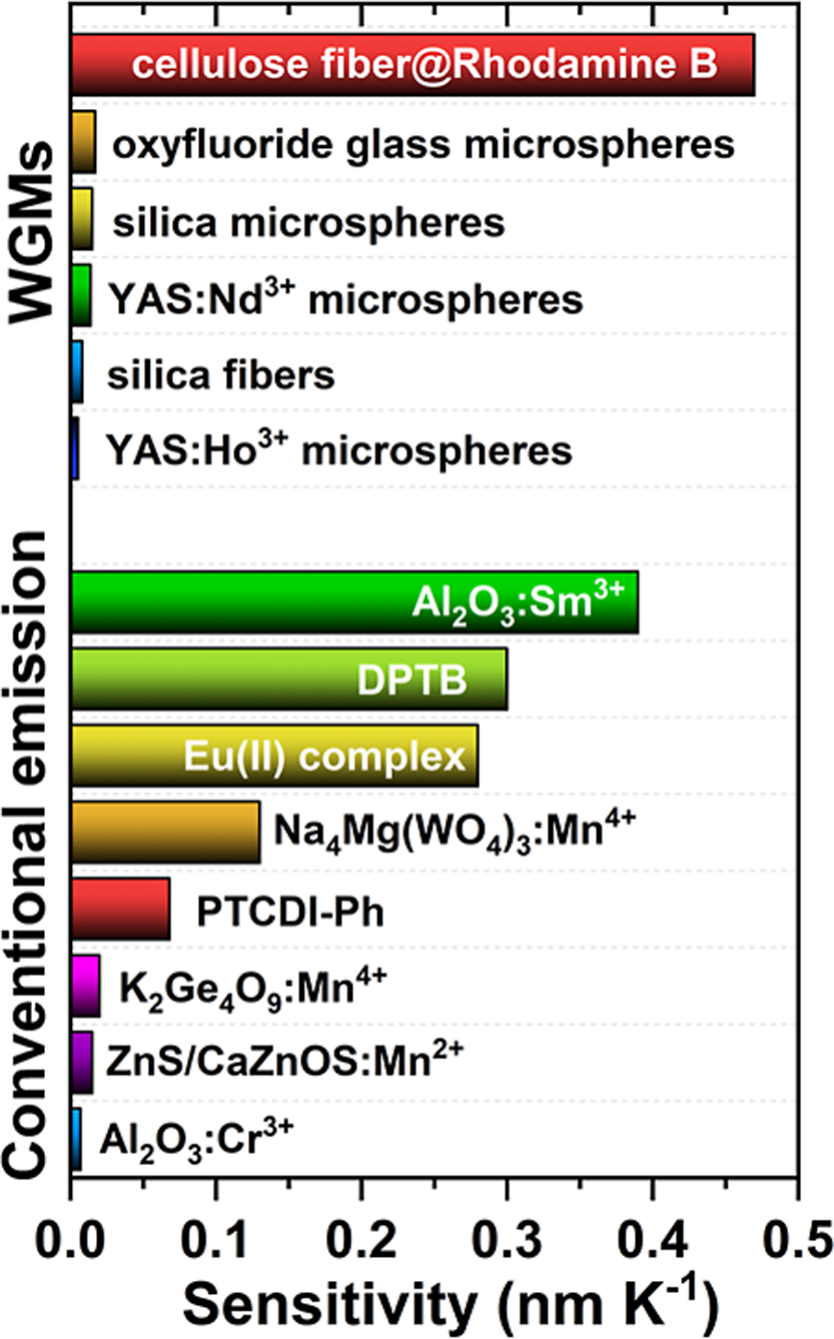
Comparison of the maximum absolute sensitivity values for WGMs and emission band shifts for various, most sensitive luminescent thermometers reported in the literature.^39,43,55,70–79^

## 4 Conclusions

Here, we developed a unique optical thermometer based on the spectral shift of the narrow emission lines originating from resonant WGM in the organic dye-doped cellulose fibers, which exhibits superior sensitivity, over 27 times higher than that for temperature sensors based on the same effects. Cellulose fibers doped with Rhodamine B were obtained by dry-wet spinning methods with *N*-methylmorpholine N-oxide (NMMO). The morphology and structure of Rhodamine B-doped cellulose fibers were investigated using XRD and SEM, indicating a uniform distribution of fiber diameter (≈16 μm). The optical properties of the produced fibers were examined by absorption and emission spectroscopy, confirming that the cellulose fibers were successfully functionalized with the Rhodamine B molecules, which were homogeneously incorporated into their structure. Using a confocal microscope setup, upon a 532 nm laser excitation at the edge of the modified cellulose fibers, they reveal sharp emission peaks, whispering gallery modes (WGMs), superimposed on the regular Rhodamine B spectra, due to the cylindrical shape of the fibers, allowing total internal reflection of the light and free propagation of the resonant modes inside them (resonating in a cavity). We have used the change of the thermo-optic coefficient and minor temperature-induced expansion of the cellulose fibers to observe the spectral shift of the WGMs under variable temperature conditions. Rhodamine B-doped cellulose fibers were used for temperature sensing with an exceptional, giant shift of the WGMs with the maximum *S*_a_ around ∼0.47 nm K^–1^ and *S*_r_ of ∼0.084% K^–1^, which are the highest values reported for such kinds of optical thermometers based on the WGMs, guaranteeing the best sensing performance for the developed microresonators so far. Additionally, the thermal sensitivity of the WGM shift is twice that of the highest spectral shifts reported for regular organic and inorganic luminescent thermometers developed to date. The obtained sensitivity values, together with very good temperature resolution (δ*T*) of 0.14–0.17 K, make the performance of the developed optical thermometer quite impressive. This research investigates the potential of biocompatible cellulose fibers and safe organic dyes for contactless temperature detection within the physiological range, enabling potential applications in living organisms. From the bioapplication point of view, our approach shows an alternative to potentially harmful or hazardous use of nanoparticles based on heavy metals or lanthanides. Furthermore, our findings open new horizons in ultrasensitive remote temperature sensing by developing the previously underestimated optical resonance effects of WGMs in organic polymer microresonators as cellulose fibers.

## Supplementary Material



## Notes

Additional analysis: simulated FSR, cycling, time-dependent WGM emission spectra, second run of temperature-dependent WGMs, and experimentally determined resolution (PDF)

## References

[1] Roriz P., Silva S., Frazão O., Novais S. (2020). Optical Fiber Temperature
Sensors and Their Biomedical Applications. Sensors.

[2] Medina-Aguilar G., Lozano-Gorrín A. D., Lavín V., Hernández-Rodríguez M. A. (2024). Expanding the Toolbox for Industrial
Luminescent Primary Thermometry: Er3+-Doped SrMoO4. J. Alloys Compd..

[3] Rovera A., Tancau A., Boetti N., Dalla Vedova M. D. L., Maggiore P., Janner D. (2023). Fiber Optic Sensors for Harsh and
High Radiation Environments in Aerospace Applications. Sensors.

[4] Jurga N., Runowski M., Grzyb T. (2024). Lanthanide-Based
Nanothermometers
for Bioapplications: Excitation and Temperature Sensing in Optical
Transparency Windows. J. Mater. Chem. C.

[5] Brites C. D. S., Marin R., Suta M., Carneiro Neto A. N., Ximendes E., Jaque D., Carlos L. D. (2023). Spotlight
on Luminescence
Thermometry: Basics, Challenges, and Cutting-Edge Applications. Adv. Mater..

[6] Szymczak M., Jaśkielewicz J., Runowski M., Xue J., Mahlik S., Marciniak L. (2024). Highly-Sensitive,
Tri-Modal Luminescent Manometer Utilizing
Band-Shift, Ratiometric and Lifetime-Based Sensing Parameters. Adv. Funct. Mater..

[7] Zheng T., Sójka M., Woźny P., Martín I. R., Lavín V., Zych E., Lis S., Du P., Luo L., Runowski M. (2022). Supersensitive Ratiometric Thermometry and Manometry
Based on Dual-Emitting Centers in Eu2+/Sm2+ -Doped Strontium Tetraborate
Phosphors. Adv. Opt. Mater..

[8] Runowski M., Shyichuk A., Tymiński A., Grzyb T., Lavín V., Lis S. (2018). Multifunctional Optical
Sensors for Nanomanometry and Nanothermometry:
High-Pressure and High-Temperature Upconversion Luminescence of Lanthanide-Doped
Phosphates-LaPO4/YPO4:Yb3+–Tm3+. ACS
Appl. Mater. Interfaces.

[9] Woźny P., Soler-Carracedo K., Stopikowska N., Martín I. R., Runowski M. (2023). Structure-Dependent Luminescence of Eu 3+-Doped Strontium
Vanadates Synthesized with Different V: Sr Ratios – Application
in WLEDs and Ultra-Sensitive Optical Thermometry. J. Mater. Chem. C.

[10] Park C. H., Kang Y. K., Im S. S. (2004). Biodegradability of Cellulose Fabrics. J. Appl. Polym. Sci..

[11] Malis D., Jeršek B., Tomšič B., Štular D., Golja B., Kapun G., Simončič B. (2019). Antibacterial
Activity and Biodegradation of Cellulose Fiber Blends with Incorporated
ZnO. Materials.

[12] Forsberg D. C. R., Bengtsson J., Hollinger N., Kaldéus T. (2024). Towards Sustainable
Viscose-to-Viscose Production: Strategies for Recycling of Viscose
Fibres. Sustainability.

[13] Chen J., Xu J., Wang K., Cao X., Sun R. (2016). Cellulose Acetate Fibers
Prepared from Different Raw Materials with Rapid Synthesis Method. Carbohydr. Polym..

[14] Szczeszak A., Skwierczyńska M., Przybylska D., Runowski M., Śmiechowicz E., Erdman A., Ivashchenko O., Grzyb T., Kulpiński P., Olejnik K. (2022). Functionalization of Cellulose Fibers and Paper with
Lanthanide-Based Luminescent Core/Shell Nanoparticles Providing 3-Level
Protection for Advanced Anti-Counterfeiting Purposes. Mater. Des..

[15] Camacho-Berríos A., Suárez O. M. (2023). Sputtering
Deposition under Limited Adatom Mobility:
An Effective Method to Prepare a SERS Substrate Based on Ag@ZnO Composite
Deposited onto Electrospun Cellulose Acetate Fibers. Appl. Phys. A: Mater. Sci. Process..

[16] Olsson C., Westman G. (2013). Wet Spinning of Cellulose
from Ionic Liquid Solutions-Viscometry
and Mechanical Performance. J. Appl. Polym.
Sci..

[17] Clarkson C.
M., Youngblood J. P. (2018). Dry-Spinning
of Cellulose Nanocrystal/Polylactic Acid
Composite Fibers. Green Mater..

[18] Skwierczyńska M., Woźny P., Runowski M., Perzanowski M., Kulpiński P., Lis S. (2020). Bifunctional Magnetic-Upconverting
Luminescent Cellulose Fibers for Anticounterfeiting Purposes. J. Alloys Compd..

[19] Skwierczyńska M., Runowski M., Kulpiński P., Lis S. (2019). Modification of Cellulose
Fibers with Inorganic Luminescent Nanoparticles Based on Lanthanide
(III) Ions. Carbohydr. Polym..

[20] Skwierczyńska M., Runowski M., Goderski S., Szczytko J., Rybusiński J., Kulpiński P., Lis S. (2018). Luminescent–Magnetic Cellulose
Fibers, Modified with Lanthanide-Doped Core/Shell Nanostructures. ACS Omega.

[21] Smiechowicz E., Niekraszewicz B., Kulpinski P. (2021). Optimisation
of AgNP Synthesis in
the Production and Modification of Antibacterial Cellulose Fibres. Materials.

[22] Kulpinski P., Erdman A., Namyslak M., Fidelus J. D. (2012). Cellulose Fibers
Modified by Eu 3+-Doped Yttria-Stabilized Zirconia Nanoparticles. Cellulose.

[23] Kasaei P., Karami N., Keyvan Rad J., Sanjabi S., Mahdavian A. R. (2022). Modified
Cellulose Paper with Photoluminescent Acrylic Copolymer Nanoparticles
Containing Fluorescein as PH-Sensitive Indicator. Carbohydr. Polym..

[24] Zhou G., Zhang Z., Meng Z., Qian C., Li M., Wang Z., Yang Y. (2023). A Highly Specific
Chalcone Derivative
Grafted Ethylcellulose Fluorescent Probe for Rapid and Sensitive Detection
of Al3+ in Actual Environmental and Food Samples. Int. J. Biol. Macromol..

[25] Li C., He Y., Zhang J., Mu J., Wang J., Cao M., Nawaz H., Chen S., Xu F. (2024). Cellulose-Based Colorimetric/Ratiometric
Fluorescence Sensor for Visual Detecting Amines and Anti-Counterfeiting. Carbohydr. Polym..

[26] Li Z. C., Li D. W., Liu Z. H., Wu L. P., lv X. S. (2024). Visualization
of Latent Fingerprints Based on Composites of Dye-Doped Cellulose
with Red Emissive Fluorescence. Cellulose.

[27] Subaihi A., Al-Qahtani S. D., Attar R. M. S., Alkhamis K., Alzahrani H. K., Alhasani M., El-Metwaly N. M. (2022). Preparation of Fluorescent Cotton
Fibers with Antimicrobial Activity Using Lanthanide-Doped Pigments. Cellulose.

[28] Liu H., Wang Y., Li H., Wang Z., Xu D. (2013). Luminescent
Rhodamine B Doped Core–Shell Silica Nanoparticle Labels for
Protein Microarray Detection. Dyes Pigm..

[29] Okano K., Taguchi M., Fujiki M., Yamashita T. (2011). Circularly
Polarized Luminescence of Rhodamine B in a Supramolecular Chiral Medium
Formed by a Vortex Flow. Angew. Chem..

[30] Tomazio N. B., Boni L. D., Mendonca C. R. (2017). Low Threshold
Rhodamine-Doped Whispering
Gallery Mode Microlasers Fabricated by Direct Laser Writing. Sci. Rep..

[31] Martinez-Quijada J., Ma T., Hall G. H., Reynolds M., Sloan D., Caverhill-Godkewitsch S., Glerum D. M., Sameoto D., Elliott D. G., Backhouse C. J. (2015). Robust
Thermal Control for CMOS-Based Lab-on-Chip Systems. J. Micromech. Microeng..

[32] Qiu S., Chu H., Zou Y., Xiang C., Zhang H., Sun L., Xu F. (2016). Thermochemical
Studies of Rhodamine B and Rhodamine 6G by Modulated
Differential Scanning Calorimetry and Thermogravimetric Analysis. J. Therm. Anal. Calorim..

[33] Chen Y., Yin Y., Ma L., Schmidt O. G. (2021). Recent
Progress on Optoplasmonic
Whispering-Gallery-Mode Microcavities. Adv.
Opt. Mater..

[34] Paz-Buclatin F., Ríos S., Martín I. R., Martin L. L. (2019). Fluorescence Intensity
Ratio and Whispering Gallery Mode Techniques in Optical Temperature
Sensors: Comparative Study. Opt. Mater. Express.

[35] Reynolds T., Riesen N., Meldrum A., Fan X., Hall J. M. M., Monro T. M., François A. (2017). Fluorescent
and Lasing Whispering
Gallery Mode Microresonators for Sensing Applications. Laser Photonics Rev..

[36] Duong
Ta V., Chen R., Ma L., Jun Ying Y., Dong Sun H. (2013). Whispering
Gallery Mode Microlasers and Refractive Index Sensing Based on Single
Polymer Fiber. Laser Photonics Rev..

[37] Labrador-Páez L., Soler-Carracedo K., Hernández-Rodríguez M., Martín I. R., Carmon T., Martin L. L. (2017). Liquid Whispering-Gallery-Mode
Resonator as a Humidity Sensor. Opt. Express.

[38] Toropov N., Cabello G., Serrano M. P., Gutha R. R., Rafti M., Vollmer F. (2021). Review of Biosensing with Whispering-Gallery
Mode Lasers. Light: Sci. Appl..

[39] Walo-Martín D., Paz-Buclatin F., Ríos S., Martín I. R., Martin L. L., Ródenas A., Sigaev V. N., Savinkov V. I., Shakhgildyan G. Y. (2021). Temperature
Sensing with Nd3+ Doped YAS Laser Microresonators. Appl. Sci..

[40] Runowski M., Woźny P., Stopikowska N., Martín I. R., Lavín V., Lis S. (2020). Luminescent Nanothermometer Operating
at Very High Temperature—Sensing up to 1000 K with Upconverting
Nanoparticles (Yb3/Tm3+). ACS Appl. Mater. Interfaces.

[41] Runowski M., Woźny P., Martín I. R., Lavín V., Lis S. (2019). Praseodymium Doped
YF3:Pr3+ Nanoparticles as Optical Thermometer
Based on Luminescence Intensity Ratio (LIR) – Studies in Visible
and NIR Range. J. Lumin..

[42] Stopikowska N., Runowski M., Skwierczyńska M., Lis S. (2021). Improving
Performance of Luminescent Nanothermometers Based on Non-Thermally
and Thermally Coupled Levels of Lanthanides by Modulating Laser Power. Nanoscale.

[43] de
Sousa-Vieira L., Ríos S., Martín I. R., García-Rodríguez L., Sigaev V. N., Savinkov V. I., Yu Shakhgildyan G. (2018). Whispering Gallery Modes in a Holmium Doped Glass Microsphere:
Temperature Sensor in the Second Biological Window. Opt. Mater..

[44] Nunzi
Conti G., Chiasera A., Ghisa L., Berneschi S., Brenci M., Dumeige Y., Pelli S., Sebastiani S., Feron P., Ferrari M., Righini G. C. (2006). Spectroscopic and
Lasing Properties of Er3+-Doped Glass Microspheres. J. Non-Cryst. Solids.

[45] Savarese M., Aliberti A., De Santo I., Battista E., Causa F., Netti P. A., Rega N. (2012). Fluorescence
Lifetimes and Quantum
Yields of Rhodamine Derivatives: New Insights from Theory and Experiment. J. Phys. Chem. A.

[46] Zhang S., Zhai T., Cui L., Shi X., Ge K., Liang N., Hayat A. (2021). Tunable Wgm Laser Based on the Polymer
Thermo-Optic Effect. Polymers.

[47] Lin G., Chembo Y. K. (2019). (INVITED) Monolithic Total Internal Reflection Resonators
for Applications in Photonics. Opt. Mater.:
X.

[48] Oh S. Y., Yoo D. I., Shin Y., Kim H. C., Kim H. Y., Chung Y. S., Park W. H., Youk J. H. (2005). Crystalline Structure
Analysis of Cellulose Treated with Sodium Hydroxide and Carbon Dioxide
by Means of X-Ray Diffraction and FTIR Spectroscopy. Carbohydr. Res..

[49] Holder C. F., Schaak R. E. (2019). Tutorial on Powder X-Ray Diffraction
for Characterizing
Nanoscale Materials. ACS Nano.

[50] Ji R., Zhao Z., Yu X., Chen M. (2019). Determination of Rhodamine
B in Capsicol Using the First Derivative Absorption Spectrum. Optik.

[51] Christie, R. M. Fluorescent Dyes. In Handbook of Textile and Industrial Dyeing; Woodhead Publishing, 2011; Vol. 1, pp 562–587.

[52] Zhang X.-F., Zhang Y., Liu L. (2014). Fluorescence Lifetimes and Quantum
Yields of Ten Rhodamine Derivatives: Structural Effect on Emission
Mechanism in Different Solvents. J. Lumin..

[53] Sugiarto I. T., Isnaeni, Putri K. Y. (2017). Analysis
of Dual Peak Emission from Rhodamine 6G Organic Dyes Using Photoluminescence. J. Phys.: Conf. Ser..

[54] Burghardt T. P., Lyke J. E., Ajtai K. (1996). Fluorescence Emission and Anisotropy
from Rhodamine Dimers. Biophys. Chem..

[55] Soler-Carracedo K., Ruiz A., Martín I. R., Lahoz F. (2019). Luminescence Whispering
Gallery Modes in Ho3+ Doped Microresonator Glasses for Temperature
Sensing. J. Alloys Compd..

[56] Goswami P., Blackburn R. S., El-Dessouky H. M., Taylor J., White P. (2009). Effect of
Sodium Hydroxide Pre-Treatment on the Optical and Structural Properties
of Lyocell. Eur. Polym. J..

[57] Savchenkov A. A., Matsko A. B., Ilchenko V. S., Solomatine I., Seidel D., Maleki L. (2008). Tunable Optical Frequency
Comb with
a Crystalline Whispering Gallery Mode Resonator. Phys. Rev. Lett..

[58] Sedlmeir F., Zeltner R., Leuchs G., Schwefel H. G. L. (2014). High-Q MgF_2
Whispering Gallery Mode Resonators for Refractometric Sensing in Aqueous
Environment. Opt. Express.

[59] Soler-Carracedo K., Ruiz A., Ríos S., de Armas-Rillo S., Martín L. L., Hohmann M., Martín I. R., Lahoz F. (2025). Stretching the Limits of Refractometric Sensing in Water Using Whispering-Gallery-Mode
Resonators. Chemosensors.

[60] Soler-Carracedo K., Martin I. R., Runowski M., Martín L. L., Lahoz F., Lozano-Gorrín A. D., Paz-Buclatin F. (2020). Luminescent
Nd 3+-Based Microresonators Working as Optical Vacuum Sensors. Adv. Opt. Mater..

[61] Sultanova N. G., Kasarova S. N., Nikolov I. D. (2013). Characterization of Optical Properties
of Optical Polymers. Opt. Quantum Electron..

[62] Wang C., Zhang X., Ma J., Xie K., Zhang J., Hu Z. (2022). Temperature Sensitivity of Polymer
Fiber Microlasers. Photonic Sens..

[63] Guan F., Chen S., Yao J., Zheng W., Wang H. (2016). ZnS/Bacterial
Cellulose/Epoxy Resin (ZnS/BC/E56) Nanocomposites with Good Transparency
and Flexibility. J. Mater. Sci. Technol..

[64] Sultanova N., Kasarova S., Nikolov I. (2013). Characteristics
of Optical Polymers
in the Design of Polymer and Hybrid Optical. Bulg. J. Phys..

[65] Labrador-Páez L., Pedroni M., Speghini A., García-Solé J., Haro-González P., Jaque D. (2018). Reliability of Rare-Earth-Doped Infrared
Luminescent Nanothermometers. Nanoscale.

[66] Slavík R., Numkam Fokoua E. R., Bukshtab M., Chen Y., Bradley T. D., Sandoghchi S. R., Petrovich M. N., Poletti F., Richardson D. J. (2019). Demonstration
of Opposing Thermal Sensitivities in Hollow-Core Fibers with Open
and Sealed Ends. Opt. Lett..

[67] Guan G., Arnold S., Otugen M. V. (2006). Temperature Measurements Using a
Microoptical Sensor Based on Whispering Gallery Modes. AIAA J..

[68] Brites C. D. S., Balabhadra S., Carlos L. D. (2019). Lanthanide-Based Thermometers: At
the Cutting-Edge of Luminescence Thermometry. Adv. Opt. Mater..

[69] Zhou J., del Rosal B., Jaque D., Uchiyama S., Jin D. (2020). Advances and
Challenges for Fluorescence Nanothermometry. Nat. Methods.

[70] Szymczak M., Piotrowski W. M., Woźny P., Runowski M., Marciniak L. (2024). A Highly Sensitive
Lifetime-Based Luminescent Manometer and Bi-Functional Pressure-Temperature
Sensor Based on a Spectral Shift of the R-Line of Mn4+ in K2Ge4O9. J. Mater. Chem. C.

[71] Feng J., Tian K., Hu D., Wang S., Li S., Zeng Y., Li Y., Yang G. (2011). A Triarylboron-Based
Fluorescent Thermometer: Sensitive Over a Wide Temperature Range. Angew. Chem., Int. Ed..

[72] Ratajczyk P., Sobczak S., Woźny P., Wcisło A., Poręba T., Katrusiak A. (2023). Unlocking the Sensing Potential of
Phenyl-Substituted Perylene Diimides under Extreme Conditions. J. Mater. Chem. C.

[73] Ćirić A., Stojadinović S., Ristić Z., Zeković I., Kuzman S., Antić Z. ˇ., Dramićanin M. D. (2021). Supersensitive
Sm2+-Activated Al2O3 Thermometric Coatings for High-Resolution Multiple
Temperature Read-Outs from Luminescence. Adv.
Mater. Technol..

[74] Amarasinghe D. K., Rabuffetti F. A. (2019). Bandshift
Luminescence Thermometry Using Mn4+: Na4Mg­(WO4)­3
Phosphors. Chem. Mater..

[75] Diaz-Rodriguez R. M., Gálico D. A., Chartrand D., Suturina E. A., Murugesu M. (2022). Toward Opto-Structural
Correlation to Investigate Luminescence Thermometry in an Organometallic
Eu­(II) Complex. J. Am. Chem. Soc..

[76] Rekhi S., Dubrovinsky L., Saxena S. (1999). Temperature-Induced Ruby Fluorescence
Shifts up to a Pressure of 15 GPa in an Externally Heated Diamond
Anvil Cell. High Temp. - High Pressures.

[77] Zheng T., Runowski M., Martín I. R., Soler-Carracedo K., Peng L., Skwierczyńska M., Sójka M., Barzowska J., Mahlik S., Hemmerich H., Rivera-López F., Kulpiński P., Lavín V., Alonso D., Peng D. (2023). Mechanoluminescence and Photoluminescence
Heterojunction for Superior Multimode Sensing Platform of Friction,
Force, Pressure, and Temperature in Fibers and 3D-Printed Polymers. Adv. Mater..

[78] Soler-Carracedo K., Estévez-Alonso P., Martin I. R., Rios S. (2021). Improving
the Sensitivity of WGM Pressure Sensors with Oxyfluoride Glass Microspheres. J. Lumin..

[79] Rivera-Perez E., Villegas I. L., Diez A., Andres M. V., Cruz J. L., Rodriguez-Cobos A. (2013). Measurement
of Pump-Induced Temperature Increase in
Doped Fibers Using Whispering-Gallery Modes. IEEE Photonics Technol. Lett..

