# Supplementary material for: Breakthrough in Luminescence Thermometry—Supersensitive Emission Line Shift of Whispering Gallery Modes in Rhodamine B‑Doped Cellulose Fiber Microresonators
Source: ACS Appl Mater Interfaces. 2025 Oct 1;17(43):59737–46. doi: 10.1021/acsami.5c12765 (PMC12581120; doi:10.1021/acsami.5c12765)
Supplement: Supplementary file 1 [file am5c12765_si_001.pdf]

Supporting Information

Breakthrough in Luminescence Thermometry - Supersensitive Emission Line Shift of Whispering Gallery Modes in Rhodamine B-doped Cellulose Fiber Microresonators

Przemysław Woźny,[a,*] Kevin Soler-Carracedo,[a] Małgorzata Skwierczyńska,[a] Inocencio R. Martin,[b] Piotr Kulpiński,[c] Marcin Runowski [a]

[a] *Faculty of Chemistry, Adam Mickiewicz University, Uniwersytetu Poznańskiego 8, Poznań 61-614, Poland*

[b] *Departamento de Física, Instituto de Materiales y Nanotecnología (IMN), Universidad de La Laguna, San Cristóbal de La Laguna E-38200, Santa Cruz de Tenerife, Spain*

[c] *Department of Mechanical Engineering, Informatics and Chemistry of Polymer Materials, Lodz University of Technology, Żeromskiego 116, Łódź 90-924, Poland*

Corresponding author: Dr. P. Woźny,

*__E-mail__: (P.W.) przemyslaw.wozny@amu.edu.pl

The Rhodamine B is well known xanthene-based organic dye with broad emission band in range λ ≈ 550-700 nm, proposed as laser dye and as marker in bioimaging. We have present photoluminescence mechanism in Fig. S1b. Upon UV excitation, Rhodamine B absorb the photon and undergoes a π → π* transition in which an electron is promoted from the HOMO (Highest Occupied Molecular Orbital) of the ground singlet state (S_0) to the LUMO (Lowest Unoccupied Molecular Orbital), populating higher excited singlet state (S_2). Next, non-radiative relaxation from S_2 to S_1 singlet state occur. Once the molecule is in the relaxed S1 state, radiative decay to the ground state S_0 occur, causing photoluminescence with visible emission centered at λ ~ 600 nm. According to Kasha's rule, fluorescence emission does not occur directly from these higher states but only from the lowest excited singlet state (S_1). This is because ultrafast non-radiative processes – such as internal system conversion (ISC) and non-radiative relaxation (NR) to the lowest energy level of S_1 on a picosecond timescale. Since part of the excitation energy is lost during ISC and NR, the emitted photon has lower energy (longer wavelength) than the absorbed one, leading to the characteristic Stokes shift observed in Rhodamine B (or other organic dyes). Additionally, the excited singlet state can undergo competing nonradiative pathways, such as intersystem crossing (ISC) to the triplet state (T_1),

which may reduce the photoluminescence quantum yield at the expense of phosphorescence from T_1 to T_2 energy level. In our research we don't observe phosphorescence from Rhodamine B, the fluorescence pathway is dominant, which explains its strong emission efficiency.

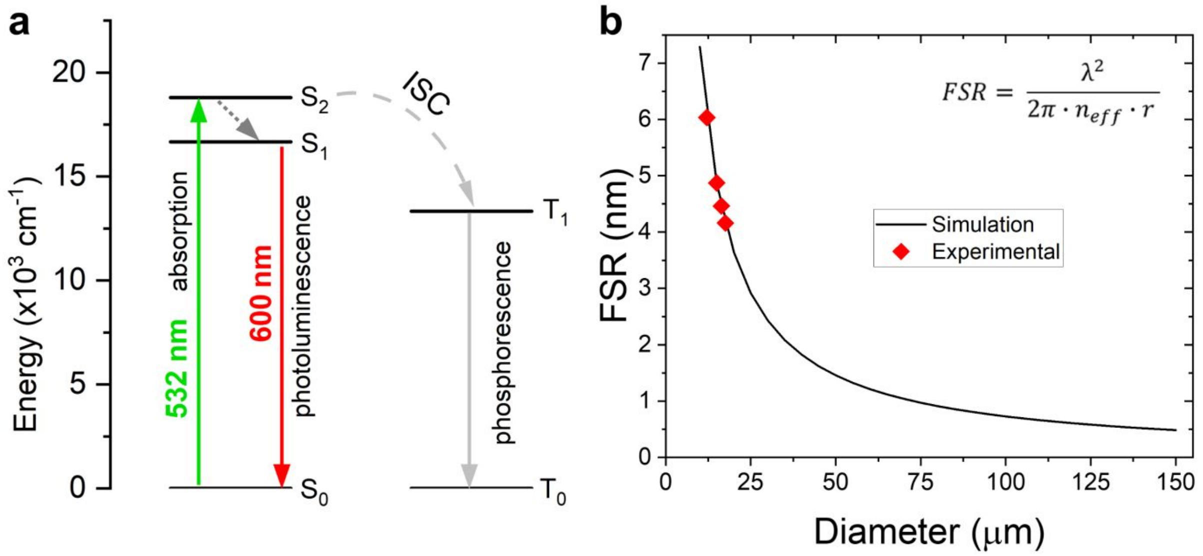

Figure S1. Luminescence mechanism of the Rhodamine B (a), Simulated evolution of the FSR values as a function of the fibers diameter (black curve), overlaid with the obtained experimental data points (red rhombi) (b).

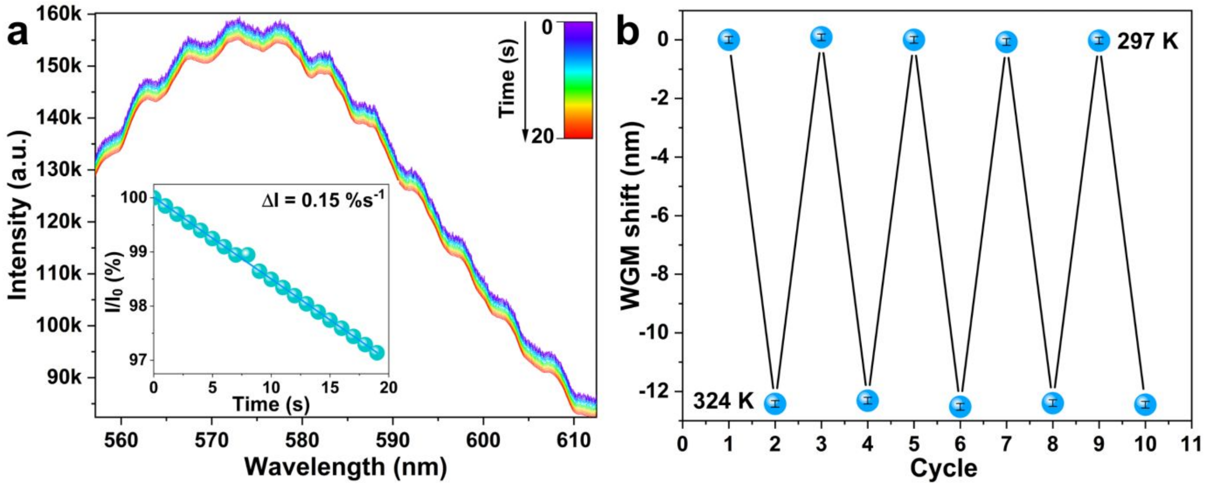

Figure S2. WGMs emission spectra recorded over 20 s under continous 532 nm laser irradiation (a); Average displacement of the selected WGMs in heating-cooling cycles between 297 and 324 K (b).

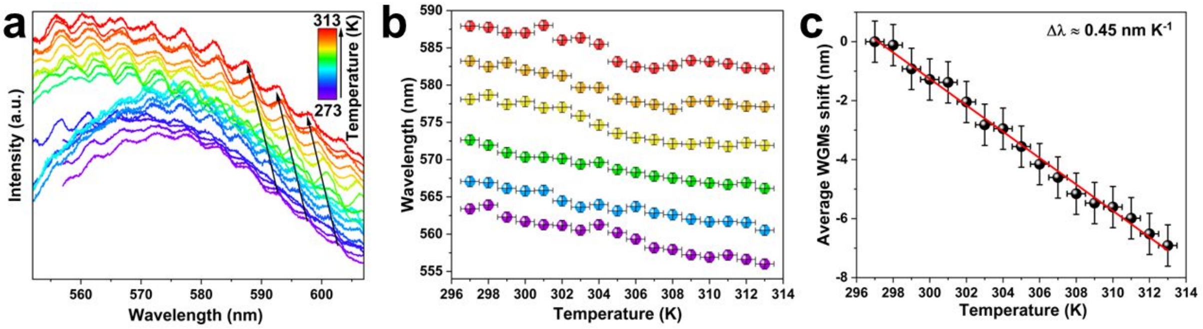

Figure S3. Emission spectra measured at different temperature values with narrow WGMs and their thermal shift (a); the calculated position of the selected WGMs as a function of temperature for second cellulose fibers@Rhodamine B (b); average WGMs shift for second fiber (c).

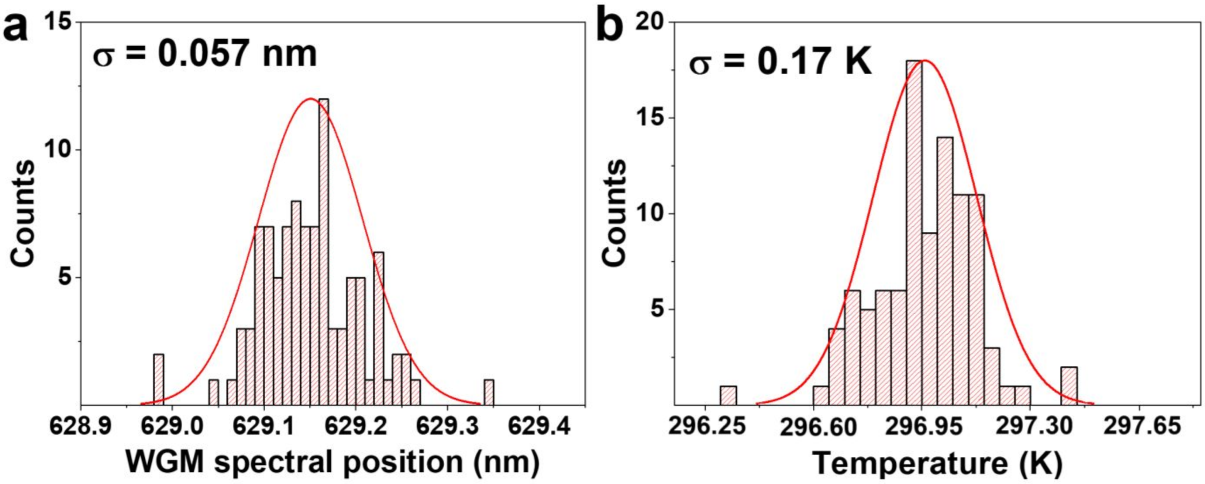

Figure S4. Histograms obtained based on the recorded series of 100 emission spectra, showing spectral resolution of the WGM mode at ≈629 nm (a) and the corresponding temperature values (b), calculated using calibration curve from Eq. 5, with their respective standard deviation (σ) values; the σ represents the experimental temperature resolution of the system.

Table. S1 List of maximum relative and absolute sensitivities of different WGM-based sensors.

Max S_r (% nm K^{-1})	Max S_a (nm K^{-1})	Matrix	Ref.
8.4×10^{-2}	4.5×10^{-1}	Cellulose fiber	This work
2.0×10^{-3}	1.5×10^{-2}	Silica microspheres	[1]
2.0×10^{-3}	1.7×10^{-2}	Oxyfluoride glass microsphere	[2]
1.5×10^{-3}	1.4×10^{-2}	Nd^{3+} doped yttria–alumina–silica (YAS) microsphere	[3]
8.1×10^{-4}	5.3×10^{-3}	Ho^{3+} doped yttria–alumina–silica (YAS) microsphere	[4]
6.0×10^{-4}	---	Silica microcavity	[5]
5.3×10^{-4}	8.2×10^{-3}	Silica fiber	[6]

References

(1) Soler-Carracedo, K.; Ruiz, A.; Martín, I. R.; Lahoz, F. Luminescence Whispering Gallery Modes in Ho^{3+} Doped Microresonator Glasses for Temperature Sensing. *J. Alloys Compd.* **2019**, *777*, 198–203. DOI: 10.1016/j.jallcom.2018.10.297.

(2) Paz-Buclatin, F.; Ríos, S.; Martín, I. R.; Martin, L. L. Fluorescence Intensity Ratio and Whispering Gallery Mode Techniques in Optical Temperature Sensors: Comparative Study. *Opt. Mater. Express* **2019**, *9* (10), 4126. DOI: 10.1364/ome.9.004126.

(3) Walo-Martín, D.; Paz-Buclatin, F.; Ríos, S.; Martín, I. R.; Martin, L. L.; Ródenas, A.; Sigaev, V. N.; Savinkov, V. I.; Shakhgildyan, G. Y. Temperature Sensing with Nd^{3+} Doped YAS Laser Microresonators. *Appl. Sci.* **2021**, *11* (3), 1117. DOI: 10.3390/app11031117.

(4) de Sousa-Vieira, L.; Ríos, S.; Martín, I. R.; García-Rodríguez, L.; Sigaev, V. N.; Savinkov, V. I.; Yu Shakhgildyan, G. Whispering Gallery Modes in a Holmium Doped Glass Microsphere: Temperature Sensor in the Second Biological Window. *Opt. Mater. (Amst).* **2018**, *83* (February), 207–211. DOI: 10.1016/j.optmat.2018.06.014.

(5) Carmon, T.; Yang, L.; Vahala, K. J. Dynamical Thermal Behavior and Thermal Self-Stability of Microcavities. In *Integrated Photonics Research and Applications/Nanophotonics for Information Systems*; OSA: Washington, D.C., 2005; Vol. 12, p IWE4. DOI: 10.1364/IPRA.2005.IWE4.

(6) Rivera-Perez, E.; Villegas, I. L.; Diez, A.; Andres, M. V.; Cruz, J. L.; Rodriguez-Cobos, A. Measurement of Pump-Induced Temperature Increase in Doped Fibers Using Whispering-Gallery Modes. *IEEE Photonics Technol. Lett.* **2013**, *25* (24), 2498–2500. DOI: 10.1109/LPT.2013.2288865.
